# Nodopathies in the Early Diagnosis of Axonal Forms of Guillain-Barré Syndrome

**DOI:** 10.3389/fneur.2022.902172

**Published:** 2022-05-25

**Authors:** Sara Urdiales-Sánchez, José-Ramiro González-Montaña, Ricardo Diaz-Pérez, Pablo Calvo-Calleja, María-Antonia Gutiérrez-Trueba, Javier Urdiales-Urdiales

**Affiliations:** ^1^Section of Clinical Neurophysiology, Universitary Hospital of Cabueñes, Gijón, Spain; ^2^Department of Medicine, Surgery and Anatomy Veterinary, University of León, León, Spain; ^3^Service of Clinical Neurophysiology, Universitary Hospital of León, León, Spain

**Keywords:** acute motor axonal neuropathy, nerve conduction blocks, nodopathies, nodo-paranodopathies, regional variants, Guillain-Barré syndrome

## Abstract

**Introduction:**

Guillain-Barré syndrome (GBS) has been classified into demyelinating and axonal subtypes or forms, such as acute motor axonal neuropathy (AMAN) and regional pharyngeal-cervical-brachial variant (PCBv).

**Objective:**

To study the relationship between motor nerve conduction blocks (CBs) and prognosis in AMAN and PCBv.

**Patients and Methods:**

We retrospectively analyzed six cases of AMAN and PCBv with serial nerve conduction studies (NCS) and electromyography (EMG).

**Results:**

The serial NCS (1st−2nd and 3rd week) showed, as the most constant data, a decreased amplitude of the compound muscle action potential (CMAP) in 100% of cases. CBs were present in 66.6% of cases. EMG (3rd week) showed signs of severe denervation in 33.3%. All patients were treated from the 1st−2nd week of evolution with intravenous immunoglobulins (IVIGs). Patients with CBs (1st−2nd and 3rd week), showed reversible CBs or reversible conduction failure (RCF) and complete recovery at 1 month. Patients without CBs, with persistent reduced distal CMAP amplitude (dCMAP), showed severe acute denervation due to axonal degeneration (3rd week and 1st−3rd month) and a slow recovery of several months.

**Conclusions:**

Not all axonal forms of GBS have a poor prognosis. This study of AMAN and PCBv shows that patients with CBs can have reversible CBs or RCF, and good prognosis. Patients without CBs, with persistent reduction of dCMAP amplitude decrement, have severe acute denervation, and a worse prognosis. AMAN and PCBv have a continuous spectrum ranging from CBs due to dysfunction/disruption of Nodes of Ranvier, called nodopathies, with reversible CBs or RCF and good prognosis, to axonal degeneration with worse prognosis.

## Introduction

Traditionally and currently, Guillain-Barre Syndrome (GBS) applies to a broad spectrum of acute acquired and immune-mediated inflammatory polyradiculoneuropathies, with classification generally into two major groups or forms, demyelinating forms, such as acute demyelinating polyneuropathy (AIDP) and axonal forms, such as acute motor axonal neuropathy (AMAN) and the more frequent regional pharyngeal-cervical-brachial variant (PCBv) with axonal pattern (1).

The frequency of AMAN ranges from 6–7% in the United Kingdom and Spain to 30–65% in Asia and Central and South America (1).

Acute motor axonal neuropathy is a post-infectious autoimmune process against peripheral nerve antigens, associated with the presence of antiganglioside antibodies anti-GM1, GD1a, GM1b, and GalNAc-GD1a (1–3). PCBv is associated with a heterogeneous immunological profile of anti-ganglioside, anti-GT1a, anti-GT1b, GQ1b, GD1a, and GT1b antibodies (4–6). Gangliosides contribute to the stability of proteins that maintain the binding of axon and myelin at the paranodes and of sodium channels at the nodes of Ranvier. Antibodies bind to gangliosides at nodes of Ranvier, resulting in inactivation of voltage-dependent sodium channels or may produce primary axonal degeneration (3, 7).

The diagnosis is clinical, supported by electrodiagnostic, cerebrospinal fluid (CSF), and antiganglioside antibodies. AMAN presents with acute progressive weakness, with relative symmetry in upper and lower limbs, without sensory symptoms and osteotendinous reflexes usually being preserved (1). PCBv appears with progressive oropharyngeal, facial, and cervicobrachial weakness (sometimes affecting the arms) (1, 8, 9).

Based on electrodiagnosis, AMAN was previously characterized by axonal degeneration with decreased CMAP amplitude without signs of demyelination (10, 11). Currently, motor conduction blocks (CBs) without signs of demyelination (temporal dispersion of the CMAP) are described as being, in some cases, reversible CBs or reversible conduction failure (RCF) (12). A similar finding restricted to the upper extremities is observed in the PCBv (9, 13, 14).

## Materials and Methods

We retrospectively analyzed six cases of patients with AMAN and regional axonal variant PCBv, aged 18–73 years, from the Universitary Hospital of León. The research protocol and informed consent were requested and approved by the corresponding Medical Research Ethics Committee (CEIm).

Patients began with progressive loss of strength in their extremities (4 cases), dysphagia and neck and upper limb muscles weakness (1 case), and dysarthria followed by dysphagia and bilateral facial paralysis (1 case). No sensory symptoms were detected in any of the patients. The clinical features and neurophysiological studies (serial NCS and needle EMG) findings were evaluated. Laboratory test, such as antiganglioside antibodies serologies and cerebrospinal fluid (CSF) test were analyzed.

Electrophysiological studies were carried out using a Keypoint Medtronic EMG system. Recordings were performed by standard methods using surface stimulating and recording electrodes. The MCV measurement of the median and ulnar nerves was carried out by stimulation at the wrist, elbow, axilla, and Erb's point while recording over abductor pollicis brevis (APB) and abductor digiti minimi (ADM), respectively. MCV of peroneal nerve was assessed by stimulation at the ankle and knee while recording over the extensor digitorum brevis muscle (EDB). Minimal F-wave latencies were recorded from APB and abductor hallucis muscles after stimulation at the wrist and ankle, respectively. An H-reflex was recorded over soleus muscle after tibial nerve stimulation at the popliteal fossa. The axillary nerve was stimulated at the Erb's point while recording from the deltoid muscle. The spinal accessory nerve was stimulated at the posterior triangle of the neck while recording from the trapezius muscle. The phrenic nerve conduction study was stimulated on posterior border of the sternocleidomastoid muscle and recorded 5 cm from the xiphoid process (G1) with reference electrode (G2) 16 cm away from the ipsilateral costal margin. Sensory conduction velocity (SCV) of the median and ulnar nerves was determined from digit III and V to the wrist, respectively. An antidromic SCV of the sural nerves was obtained after stimulating the midcalf. Electromyography (EMG) was recorded with concentric needle electrodes from the right deltoid, APB, tibialis anterior (TA), and facial muscles. We analyzed the duration and morphology of the motor units, the presence of spontaneous activity, and the EMG pattern at maximum voluntary effort.

Accuracy was optimized using the Rajabally et al. (15) and Uncini et al. (16) electrodiagnostic criteria for axonal forms of GBS. In summary, the first and second studies must not show AIDP features and at least one of the following in two nerves: distal compound muscle action potential (dCMAP) amplitude <80% lower limit of normal (LLN) with persistent reduction in the following studies; proximal/distal CMAP (pCMAP/dCMAP) amplitude ratio <0.7 (excluding tibial nerve) on the first test, which can be recovered without increased temporal dispersion (dCMAP duration ≤120% ULN or pCMAP/dCMAP duration ratio ≤130%); absence of isolated F waves (or persistence <20%). The term RCF is used when at least in two nerves, there is evidence of a >150% increase dCMAP amplitude without increased dCMAP duration (≤120% ULN) or pCMAP/dCMAP amplitude ratio <0.7 in the first test that improves more than 0.2 in the following tests without increase in temporal dispersion (16). According to this, the term RCF applies to CBs that recover without showing temporal dispersion, a correlate of de-remyelination.

## Results

All 6 cases had a history of previous upper respiratory tract infection or self-limited diarrhea, with an acute progressive course of weakness, without sensory symptoms (Table 1). Serial NCS showed a decreased CMAP amplitude >80% LLN in all cases, as well as at least two nerves with normal conduction velocities (CVs) and normal sensory conduction parameters. The CBs were established according to the criteria described by the American Association of Neuromuscular and Electrodiagnostic Medicine (AANEM) (17) and criteria described by Rajabally et al. (15) and Uncini et al. (16): a definite partial CB was defined as a decrease in amplitude or area >50%, with a temporal dispersion <30%. Probable partial CB was defined as an amplitude or area decrease of 30–49%, with a temporal dispersion <30%. RCF was defined according to criteria described from Uncini et al. (16).

**Table 1:** Clinical features, CSF, antiganglioside antibodies and evolution.

**Case**	**Clinical features**	**CSF and other test**	**Antiganglioside antibodies**	**Evolution Recovery**
1	Acute and progressive weakness of upper and lower limbs	Normal CSF	Negative (made on day 20th after the onset)	Good evolution. Rapid recovery. Hospital discharge after a month.
2	Acute and progressive weakness of upper and lower limbs	CSF with increased protein level	Negative (made on day 15th after the onset)	Good evolution. Rapid recovery. Hospital discharge after a month.
3	Acute and progressive weakness of upper and lower limbs	CSF with increased protein level	Anti-GM1 positive (made on day 10th after the onset)	Slow recovery lasting several months.
4	Acute and progressive weakness of upper and lower limbs	CSF with increased protein level	Anti-GM1 positive (made on day 11th after the onset)	Slow recovery lasting several months.
5	Acute dysphagia, followed by weakness of neck muscles and upper limb	Normal CSF. Cranial and medullary MRI normal	Negative (made on day 20th after the onset)	Good evolution. Rapid recovery. Hospital discharge after a month.
6	Acute dysarthria followed by dysphagia, bilateral facial paralysis, velopharyngeal and lingual paralysis and dyspnea.	Normal CSF. Cranial MRI and MRA normal	Negative (made on day 20th after the onset)	Good evolution. Rapid recovery. Hospital discharge after a month

*CSF, cerebrospinal fluid; MRI, magnetic resonance imaging; MRA, magnetic resonance angiography*.

**Figure 1:**
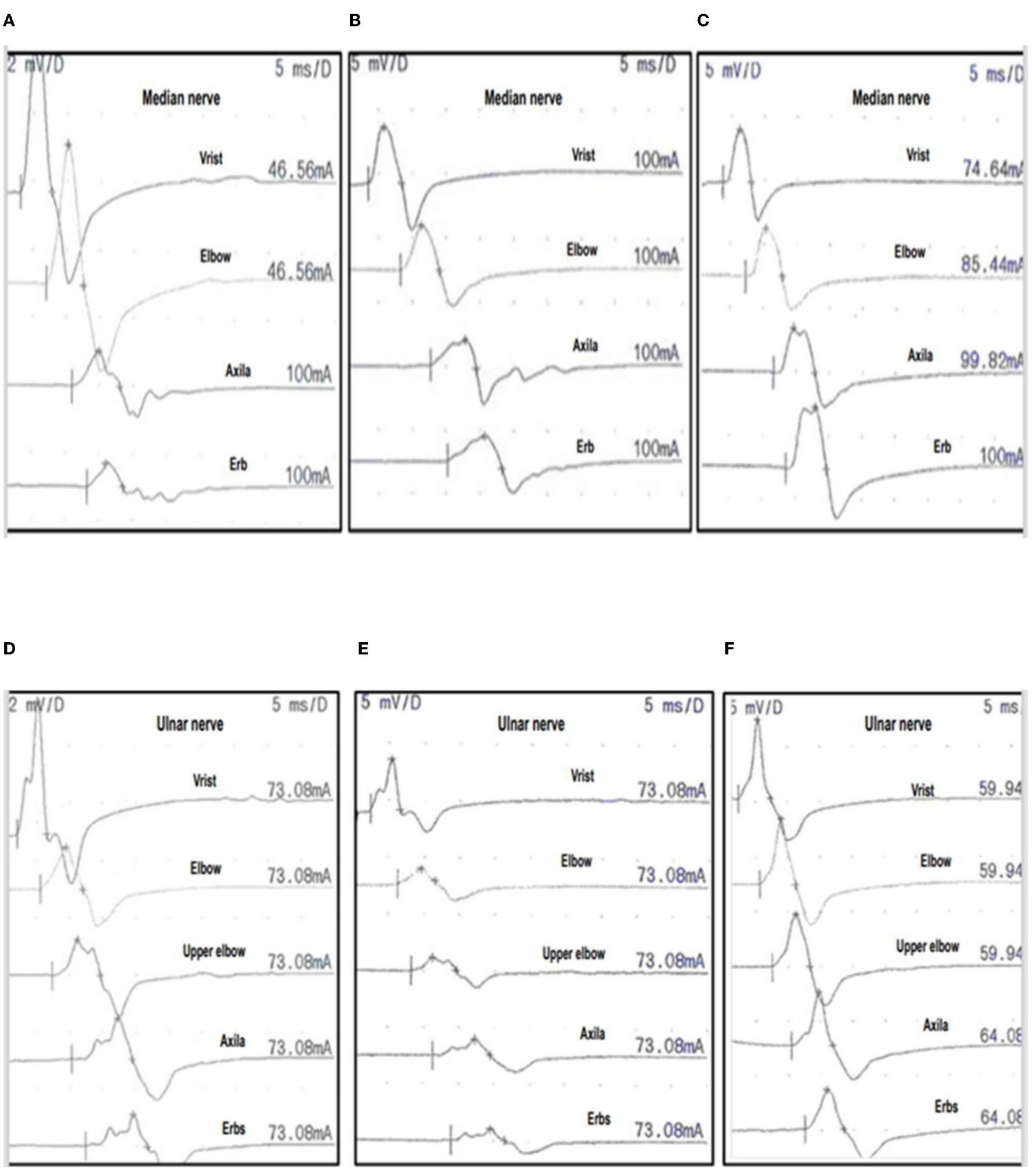
Serial nerve conduction studies (NCS) of case 1. Three serial NCS of the median nerve **(A–C)** and ulnar nerve **(D–F)** in case 1, performed at 1 week, 3 weeks, and 1 month after onset (see Tables 2, 3 for nerve conduction values). Median nerve: 1st week **(A)**, 3rd week **(B)**, 4th at 1 month **(C)** with CMAP recorded after stimulation from distal to proximal, with stimulation at wrist elbow, axilla and Erb's point, to APB muscle. Ulnar nerve: 1st week **(D)**, 3rd week **(E)**, 4th at 1 month **(F)** with CMAP recorded after distal to proximal stimulation at wrist, below elbow, above elbow, axilla and Erb's point, to ADM muscle. In the 1st and 3rd week the median and ulnar nerves show CBs or amplitude ratio pCMAP/dCMAP <0.7, and duration ratio pCMAP/dCMAP <130%. DML and MCV are preserved (Table 3). At 1 month, RCF (amplitude ratio pCMAP/dCMAP <0.7 on the first test) improves more than 0.2 in the median and ulnar nerve. Consequently, these changes are indicative of RCF. APB, abductor pollicis brevis; ADM, abductor digiti minimi; BCs: motor conduction blocks; CMAP, compound motor action potential; pCMAP, proximal CMAP; dCMAP, distal CMAP; DML, distal motor latency; VCM, motor conduction velocity.

**Figure 2:**
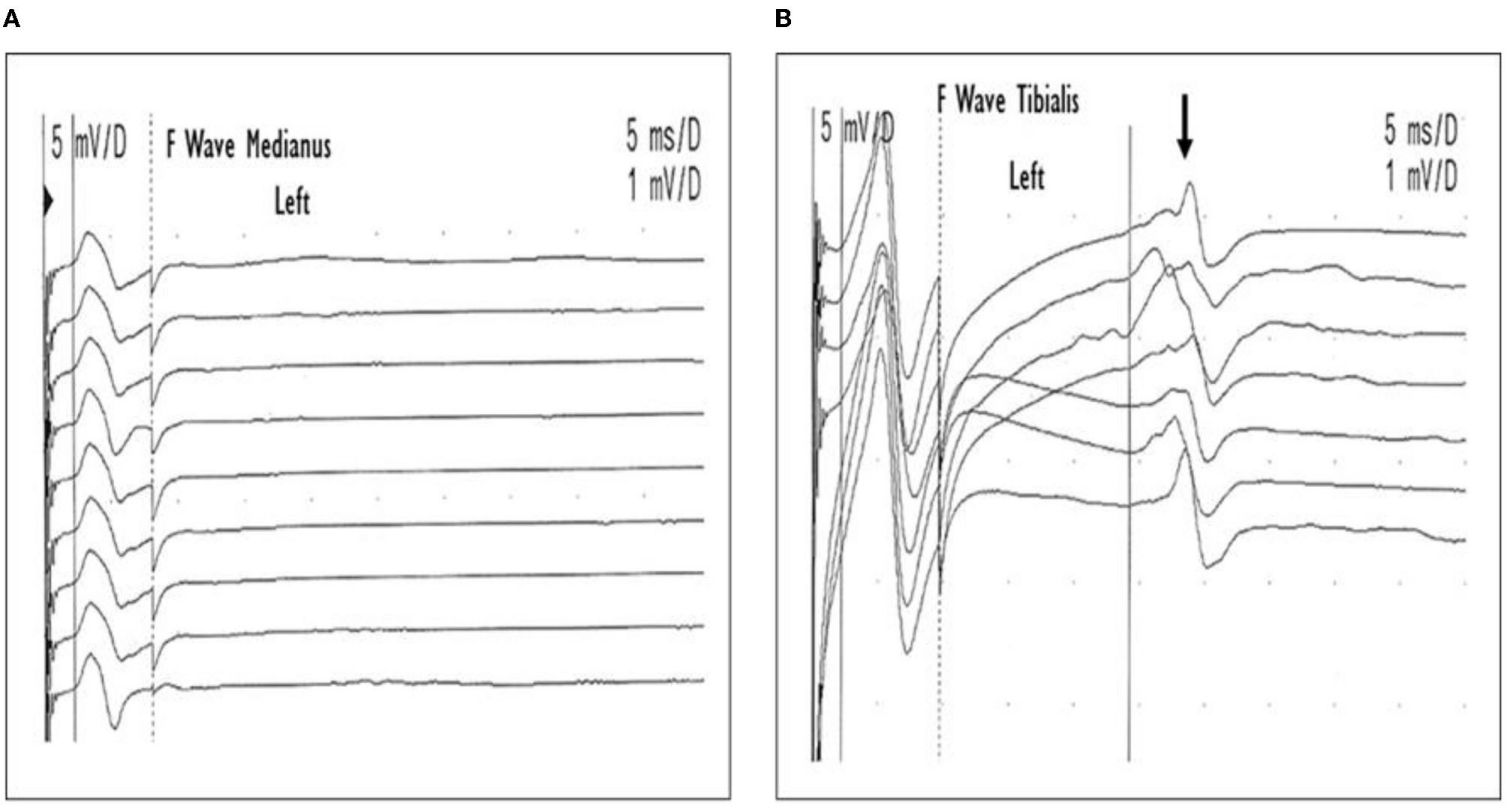
Case 5. Pharyngo-cervical-brachial variant (PCBv). F wave performed in the 2nd week. Low amplitude CMAP and absence of F waves of the left median nerve **(A)**, and normal F waves (arrow) in the left tibial nerve **(B)**.

Four cases with CBs (1st−2nd and 3rd week), one of them (PCBv) with an absence of F waves in upper limbs (2nd and 3rd week), and absent or mild signs of acute denervation (3rd week) showed reversible CBs or RCF and complete recovery in 1 month. Two cases without CBs (1st−2nd and 3rd week), with persistent reduction in dCMAP amplitude and severe signs of acute denervation due to axonal degeneration (3rd week and 1st−3rd month) showed slow recovery up to several months (Figures 1, 2 and Tables 2, 3).

**Table 2:** Nerve conduction studies (NCS) and electromyography (EMG).

**Case**	**1st NCS: 1st-2nd week**	**2nd NCS and EMG: 3rd week**	**3rd NCS and EMG: 1nd-3rd month**	**Diagnosis**
1	Decreased CMAP amplitude. Normal MCV. CBs. Preserved F-wave latencies.	Decreased CMAP amplitude. Normal MCV. CBs. Preserved F-wave latencies EMG: mild acute denervation in lower limbs.	Normal NCS. Normal F-waves. Reversible CBs or RCF. EMG: normal	Nodopathies AMAN
2	Decreased CMAP amplitude. Normal MCV. CBs. Preserved F-wave latencies.	Decreased CMAP amplitude. Normal MCV. CBs. Preserved F-wave latencies. EMG: absent acute denervation.	Normal NCS. Normal F-waves. Reversible CBs or RCF. EMG: normal	Nodopathies AMAN
3	Decreased CMAP amplitude. Normal MCV. Low F-wave persistence (<20%) with preserved latencies.	Decreased CMAP amplitude. Normal MCV. Low F-wave persistence (<20%), with preserved latencies. EMG: severe acute denervation in upper and lower limb muscles.	Persistent decreased CMAP amplitude. Normal MCV. EMG: severe acute denervation in upper and lower limb muscles.	Classic AMAN
4	Decreased CMAP amplitude. Normal MCV. Low F-wave persistence (<20%) with preserved latencies.	Decreased CMAP amplitude. Normal MCV. Low F-wave persistence (<20%), with preserved latencies. EMG: severe acute denervation of severe in upper and lower limb muscles.	Absence or persistent decrease of CMAP amplitude. Normal MCV. EMG: severe acute denervation in upper and lower limb muscles.	Classic AMAN
5	Decreased CMAP amplitude in upper limbs and Spinal accessory nerve. Normal MCV. CBs upper limb. Low F-wave persistence (<20%) with preserved latencies in the 1st week in upper limbs. Absence of F- waves in the 2nd week in upper limbs. Normal repetitive nerve stimulation.	Decreased CMAP amplitude in upper limbs and Spinal accessory nerve. Normal MCV. CBs in upper limb. Absence of F-waves in upper limbs. EMG: mild acute denervation in upper limbs muscles.	Normal NCS. Normal F-waves. Reversible CBs or RCF. EMG: normal	Nodopathies Regional AMAN (PCBv).
6	Decreased CMAP amplitude in upper limbs, both facial nerves, and right phrenic nerve. CBs in upper limb. Normal MCV. Preserved F-wave latencies.	Decreased CMAP amplitude in upper limbs, both facial nerves, and right phrenic nerve. CBs in upper limb. Normal MCV. Preserved F-wave latencies. EMG: mild acute denervation in orbicularis oris bilateral and upper limbs muscle	Normal NCS. Normal F-waves. Reversible CBs or RCF. EMG: normal.	Nodopathies Regional AMAN (PCBv).

*NCS, nerve conduction studies; CMAP, compound muscle action potential; MCV, motor conduction velocities; CBs, conduction blocks; RCF, reversible conduction failure*.

*To summarize table content, only motor NCS are indicated (sensory NCS were normal)*.

**Table 3:** Nerve conduction studies (NCS) in all patients.

**AMAN (Acute Motor Axonal Neuropathy)**																
		**Median nerve**					**Ulnar nerve**					**Peroneal nerve**				
		**CMAP-A**					**CMAP-A**					**CMAP-A**				
**Case**	**Day**	**(p/d ratio)**	**LDM**	**CMAP-D**	**VCM**	**Wave F**	**(p/d ratio)**	**DML**	**CMAP-D**	**VCM**	**Wave F**	**(p/d ratio)**	**LDM**	**CMAP-D**	**VCM**	**Wave F**
1	5	**0.7/4.7^a^ (0.1)^b^**	3.5	4.9	48	26	**1.1/4.4^a^ (0.2)^b^**	2.5	4.1	51	30	**1.4/4.1^a^ (0.3)^b^**	3.5	4.7	43	48
	21	**0.9/2.9^a^ (0.3)^b^**	3.1	4,6	53	22	**0.9/3.9^a^ (0.2)^b^**	2.2	4.5	51	31	**2.3/**6.2 **(0.1)^b^**	4.2	4.9	44	46
	31	4.8/4.1 (1.1)^c^	3.8	4.3	51	22	3.6/4.2 (0.8)^c^	2.4	5.0	60	30	6.1/11,1 (0.5)^c^	4.1	4.6	42	48
2	5	**0.5/2^a^ (0.2)^b^**	4.8	6.7	51	27	**0.2/2.1^a^ (0.09)^b^**	3.8	4.8	51	32	**2.1/3.9^a^ (0.5)^b^**	4.2	4.9	41	49
	21	**2.5/**6.1 **(0.4)^b^**	3.8	4.8	50	28	**0.4/0.8^a^ (0.5^b^)**	3.9	4.6	56	32	**2.3/3.5^a^ (0.6)^b^**	4.9	5.9	40	48
	32	5.5/6.1 (0.9)^c^	3.7	4.9	51	27	**2.3/2.8^a^ (0.8)^c^**	3.7	4.5	54	31	**3.4/2.9^a^ (1.0)^c^**	4.2	4.9	41	49
3	6	**1/1.4^a^** (0.7)	3.8	4.6	47	26	**0.5/0.7^a^ (0.7)**	3.5	4.9	55	27	**1.1/1.4^a^ (0.7)**	5.4	4.3	47	47
	22	**1.3/1.6^a^(0.8)**	3.3	4.6	48	27	**0.6/0.7^a^ (**0.8)	3	5.9	55	28	**0.5/0.7^a^ (0.7)**	4.8	4.9	47	48
	48	**0.9/1.4^a^ (0.6)**	3.4	4.7	48	29	**0.5/0.7^a^(0.7)**	3.2	5.8	49	30	**0.7/0.8 ^a^(**0.8)	6.5	4.8	47	48
4	7	**0.9/1.2^a^(0.7)**	3.1	4.2	49	29	**0.7/0.9^a^ (**0.7)	3.1	4.2	50	29	**0.9/1.2^a^ (**0.7)	3.2	4.3	45	48
	21	**0.5/0.7^a^ (0.7)**	4.8	4,4	49	30	**0.5/0.7^a^ (0.7)**	3.9	4.3	49	31	**0.1/0.1^a^ (1.0)**	4	7.2	45	49
	49	**0.1/0.2^a^ (0.5)**	4.9	4.9	48	31	**A**	**A**	**A**	**A**	**A**	**A**	**A**	**A**	**A**	**A**
**Pharyngeal-cervical-brachial variant (vPCBv)**																
	**Median nerve**			**Axillar nerve**			**Spinal accessory nerve**			**Facial nerve**			**Phrenic nerve**		
		**CMAP-A**														
**Case**	**Day**	**(p/d ratio)**	**DML**	**CMAP-D**	**MCV**	**FW**	**CMAP-A**	**DML**	**CMAP-D**	**CMAP-A**	**DML**	**CMAP-D**	**CMAP-A**	**DML**	**CMAP-A**	**DML**
5	5	**1.9/3.9^a^(0.4)^b^**	2.0	4.2	59	29	**2.2^a^**	2.6	3.9	**0.3^a^**	4.1	3.6	ND	ND	0.9	4.6
	21	**1.8/3.8^a^ (0.4)^b^**	2.1	4.1	50	**A**	**0.7^a^**	2.7	3.8	**0.3^a^**	4.2	3.7	ND	ND	0.8	4.7
	32	4.6/4.7 (0.9)^c^	2.9	4.7	54	29	4.7	2.6	3.7	4.6	4.1	3.6	ND	ND	0.9	4.6
6	6	**0.7/1.5 ^a^ (0.4)^b^**	2.6	4.3	52	27	4.5	3.2	3.1	3.9	2.1	3.2	**0.5^a^**	1.2	**0.1^a^**	3.7
	22	**0.9/1.9^a^ (0.4)^b^**	2.7	4.2	51	28	4.8	3.1	3.0	3.9	2.0	3.1	**0.7^a^**	1.3	**0.1^a^**	3.7
	39	4.5/4.8 (0.9)^c^	2.6	4.1	52	27	5.2	3.1	3.1	4.2	2.1	3.1	1.1	1.2	0.8	3.7

*A, absent; CMAP-A p/d, amplitude of compound muscle action potential, proximal/distal (mV); LDM, distal motor latency (ms); CMAP-D, CMAP duration (ms); F-wave, F-wave latency (ms); MCV, motor conduction velocity (m/s); ND, not done*.

*Abnormal motor nerve conduction parameters:*

a *dCMAP-A <80% LLN*.

b *BCs or amplitude ratio pCMAP/dCMAP <0.7 in first or second test*.

c *That improves more than 0.2 in the last test (RCF). CMAP-D (nerves, median >7.6 ms; ulnar >8.0 ms; and peroneal >8.5 ms)*.

*Bold indicates abnormal values that meet (16, 17, 30). To shorten the content of the table, only motor nerve conduction studies are indicated (sensory conduction values were normal). Reference values adapted from Chen et al. (33), Gutierrez- Rivas et al. (34), and Preston and Shapiro (35)*.

All patients were treated from the 1st to 2nd week of evolution with intravenous immunoglobulins (IVIGs), and their disabilities were assessed with the Hughes functional rating scale before treatment, 1 month and 6 months after onset. Rapid recovery was defined as an improvement of two or more points in the Hughes grade scoring system within 1 month from the beginning, and slow recovery defined as inability to walk independently (grade 3 or more) 1 month after.

## Discussion

### Usefulness of Anti-ganglioside Antibodies

Anti-GM1 and anti-GD1a antibodies are markers of axonal damage in axonal forms (AMAN), not seeing in demyelinating forms (AIDP) (18, 19). They are time-dependent, being more useful in early stages with progression to severe forms and they decrease over time (1, 2, 4–6, 20). Positive antiganglioside antibodies are considered a useful but not required aid in the diagnosis of axonal forms of GBS because they interfere with the albumin-cytological dissociation of CSF, which is also dependent on the time being requested (1, 9); this may explain the negative results in cases 1, 2, 5, and 6 (milder cases in which antiganglioside antibodies were requested at a later stage); and the positivity in cases 3 and 4 (severe cases, in which antiganglioside antibodies were requested earlier).

### The CBs and the Term RCF

When we talk about CBs, we mean CBs along the axon, having their marker in the reduction of the pCMAP amplitude and in the pCMAP/dCMAP ratio (distal CBs are described by reduced dCMAP amplitude). It had been observed that in some patients diagnosed with AMAN, the decrease in CMAP amplitude with CBs persisted over time, while others showed rapid recovery with normalization of previous abnormalities, indicating that some axonal forms may be characterized by reversible CBs or RCF (12, 21–24).

Uncini et al. (16) defined the term RCF when a pCMAP/dCMAP amplitude ratio <0.7 at the first test which improves more than 0.2 in the following trials, without temporal dispersion (pCMAP/dCMAP duration ratio ≤ 130%). The failure to distinguish between reversible CBs or RCF and demyelinating conduction block (decreased CMAP amplitude with pCMAP/dCMAP duration >130%) leads to the misclassification of AMAN patients with RCF as AIDP (12, 16).

### The Emerging Concept of Nodopathies and Nodo-Paranodopathies

It is based on the discovery of nodal and paranodal antigenic targets. Anti-ganglioside antibody-mediated neuropathies share a common pathogenic mechanism of node of Ranvier dysfunction/disruption, which can follow two different pathways: a rapid recovery of the affected node for AMAN with RCF or a progression of AMAN with axonal degeneration with slow recovery (7, 22, 25, 26). GBS has traditionally been classified into demyelinating or axonal subtype or form according to whether the myelin or axon is primarily affected. This dichotomous classification generated confusion in the electroneurophysiological diagnosis of axonal neuropathy with antiganglioside antibodies (AMAN and PCBv). To clarify this confusion, Unicini and Kuwabara (26) categorize neuropathies with antiganglioside antibodies, characterized by a common pathogenic mechanism of dysfunction/disruption at the nodes of Ranvier, called nodopathies. The AMAN axonal subtype of GBS is a prototype of nodopathy in the first phase of AMAN with CBs without signs of demyelination (temporal dispersion) and resolving rapidly (days or weeks). The advantages of the term nodopathies are that they points out directly to the site of nerve injury; resolve the paradox that an axonal form may be reversible with a good prognosis; and emphasizes the potential reversibility in neuropathies, which traditionally were thought to be characterized only by axonal degeneration, opening a therapeutic window for timely targeted treatments (26).

### The CBs and Prognosis

Not all axonal forms (AMAN and PCBv) of GBS have a poor prognosis, as was thought a few years ago. Animal models and clinical studies in AMAN show antibodies and complement deposits in the nodes, an immune process causing node elongation, deanchoring of the paranode non-compacted myelin, and conduction failure with CBs (7). In the initial stages of the immune process, the dysfunction of the nodal region is reversible; but if the immunological reaction progresses, the Ca+ influx into the axon causes axonal degeneration (25). The different forms of AMAN form a continuous pathophysiological spectrum ranging from RCF to axonal degeneration (23). CBs, especially reversible CBs, are a good prognostic factor in patients with AMAN (27). In EMG, severe acute denervation indicates axonal degeneration and a worse prognosis, whose recovery requires longer time (28).

### Early Diagnosis and Treatment of GBS

Alterations of the proximal nerve trunk with CBs in NCS are a very relevant feature in the early stages of the disease (29, 30). Since the pathophysiology of GBS is dynamic, serial NCS is necessary for the accurate classification of GBS subtypes at an early stage (12, 16, 30). Early diagnosis of GBS in the first week of evolution is difficult, but it is important to start treatment early, since 25% of patients need ventilatory support and 20% are unable to walk after 6 months (1).

### RCF and Potential Therapeutic Implications

The presence of RCF indicates, in antibody-mediated disorders, a temporary therapeutic potential. Treatments targeting conduction failure before axonal degeneration occurs may be beneficial. The presence of potentially reversible conduction failure has implications for diagnosis and prognosis and stimulates the search for targeted immunological treatments that may halt progression to axonal degeneration (31).

### Therefore, We Make a Protocol Proposal for Early Diagnosis and Treatment

Perform the first NCS of the nerve trunks, such as proximal segments, and F waves during the 1st week of evolution; if there are data of possible GBS, start treatment with IVIG; if there are no conclusive data, the NCS should be repeated during the 2nd week. Perform the following NCS and EMG in the 3rd week and at 1 month of evolution to determine the subtype or form of GBS and the presence of reversible CBs or RCF for possible nodopathies, indicating a good prognosis, and to determine the degree of acute denervation, which if severe, would indicate a worse prognosis. Early diagnosis and treatment could be essential to avoid, as far as possible, an evolution to axonal degeneration. It should be recalled that in an early stage, the GBS diagnosis can be essentially clinical supported by electrophysiological studies. Thus, treatment should be initiated as early as possible, if necessary, without the confirmatory data from electrophysiological studies.

We propose the use of the following terminology: nodo-paranodopathies in the initial phases of GBS, when CBs are observed, nodopathies if the CBs are reversible or RCF and paranodopathies if CBs with temporal dispersion of the CMAP is observed; and to continue to use the classical diagnosis AMAN in the more advanced axonal forms with axonal degeneration, and AIDP in the demyelinating forms; thus respecting the traditional and the new emerging term of nodo-paranodopathies. In our opinion, today the term nodopathy is not a diagnosis but a pathophysiological category.

The term nodopathy with RCF does apply to an early phase as it takes at least two serial NCS and few weeks from onset. In the early GBS phase, CBs without abnormal temporal dispersion in the first phase can be found also in classical AIDP, which is a demyelinating paranoid-internodopathy (32).

## Conclusion

Not all axonal forms of GBS have axonal degeneration with a poor prognosis. AMAN and PCBv have a continuous pathophysiological spectrum ranging from CBs due to dysfunction/disruption of the nodes of Ranvier, called nodopathies, with reversible CBs or RCF and good prognosis, to axonal degeneration, with a worse prognosis. According to the findings of the AMAN and PCBv studies, patients with CBs can have reversible CBs or RCF, absent or mild acute denervation and better prognosis. Patients without CBs and with persistent reduction in dCMAP amplitude have severe acute denervation due to axonal degeneration and a worse prognosis. NCS can support the early diagnosis of GBS, if we observe alteration of F waves, or CBs, which are a very relevant feature in the initial phase, sometimes necessary for serial NCS and also for the classification of GBS subtypes. The term nodopathies points directly to the site of the lesion and highlights the possible reversibility of these neuropathies that were traditionally characterized only by axonal degeneration, and stimulates research into immunological treatments with specific monoclonal antibodies.

## Data Availability Statement

The raw data supporting the conclusions of this article will be made available by the authors, without undue reservation.

## Ethics Statement

The studies involving human participants were reviewed and approved by Medical Research Ethics Committee (MREC), date 25-06-2019; number 1996.- Acute polyradiculoneuropathy. Guillain-Barré Syndrome. The importance of electromyography in the early diagnosis. Main Research: JUU from Clinical Neurophysiology. The patients/participants provided their written informed consent to participate in this study.

## Author Contributions

SU-S and JU-U: study concept and design, drafting of the initial manuscript, nerve conduction studies (NCS), electromyography (EMG), and acquisition of data. SU-S, RD-P, PC-C, and M-AG-T: literature review. SU-S, JU-U, and J-RG-M: data review, interpretation and discussion of results, and revision of the final manuscript. All authors approved the final version of the manuscript.

## Conflict of Interest

The authors declare that the research was conducted in the absence of any commercial or financial relationships that could be construed as a potential conflict of interest.

## Publisher's Note

All claims expressed in this article are solely those of the authors and do not necessarily represent those of their affiliated organizations, or those of the publisher, the editors and the reviewers. Any product that may be evaluated in this article, or claim that may be made by its manufacturer, is not guaranteed or endorsed by the publisher.

## Abbreviations

AIDP: acute inflammatory demyelinating polyneuropathy
AMAN: acute motor axonal neuropathy
CBs: nerve conduction blocks
CMAP: compound muscle action potential
CMAP-A p/d: compound muscle action amplitude proximal/distal (mV = millivolts)
CMAP-D: CMAP duration (ms = milliseconds)
CSF: cerebrospinal fluid
CV: conduction velocities
dCMAP: distal compound muscle action potential
DML: distal motor latency (ms = milliseg)
EMG: electromyography
CMAP-D: compound muscle action potential duration (ms = milliseconds)
GBS: Guillain-Barre syndrome
IVIG: intravenous immunoglobulins
LLN: lower limit of normal
MCV: motor conduction velocities
MRA: magnetic resonance angiography
NCS: nerve conduction studies
PCBv: pharyngeal-cervical-brachial variant
pCMAP: proximal compound muscle action potential
RCF: reversible conduction failure
and ULN: upper limit of normal.
